# Evaluation of lung toxicity risk with computed tomography ventilation image for thoracic cancer patients

**DOI:** 10.1371/journal.pone.0204721

**Published:** 2018-10-03

**Authors:** Masakazu Otsuka, Hajime Monzen, Kenji Matsumoto, Mikoto Tamura, Masahiro Inada, Noriyuki Kadoya, Yasumasa Nishimura

**Affiliations:** 1 Department of Medical Physics, Graduate School of Medical Science, Kindai University, Osakasayama, Japan; 2 Department of Radiation Oncology, Kindai University Faculty of Medicine, Osakasayama, Japan; 3 Department of Radiation Oncology, Tohoku University Graduate School of Medicine, Sendai, Japan; North Shore Long Island Jewish Health System, UNITED STATES

## Abstract

**Background:**

Four-dimensional computed tomography (4D-CT) ventilation is an emerging imaging modality. Functional avoidance of regions according to 4D-CT ventilation may reduce lung toxicity after radiation therapy. This study evaluated associations between 4D-CT ventilation-based dosimetric parameters and clinical outcomes.

**Methods:**

Pre-treatment 4D-CT data were used to retrospectively construct ventilation images for 40 thoracic cancer patients retrospectively. Fifteen patients were treated with conventional radiation therapy, 6 patients with hyperfractionated radiation therapy and 19 patients with stereotactic body radiation therapy (SBRT). Ventilation images were calculated from 4D-CT data using a deformable image registration and Jacobian-based algorithm. Each ventilation map was normalized by converting it to percentile images. Ventilation-based dosimetric parameters (Mean Dose, V5 [percent lung volume receiving ≥5 Gy], and V20 [percent lung volume receiving ≥20 Gy]) were calculated for highly and poorly ventilated regions. To test whether the ventilation-based dosimetric parameters could be used predict radiation pneumonitis of ≥Grade 2, the area under the curve (AUC) was determined from the receiver operating characteristic analysis.

**Results:**

For Mean Dose, poorly ventilated lung regions in the 0–30% range showed the highest AUC value (0.809; 95% confidence interval [CI], 0.663–0.955). For V20, poorly ventilated lung regions in the 0–20% range had the highest AUC value (0.774; 95% [CI], 0.598–0.915), and for V5, poorly ventilated lung regions in the 0–30% range had the highest AUC value (0.843; 95% [CI], 0.732–0.954). The highest AUC values for Mean Dose, V20, and V5 were obtained in poorly ventilated regions. There were significant differences in all dosimetric parameters between radiation pneumonitis of Grade 1 and Grade ≥2.

**Conclusions:**

Poorly ventilated lung regions identified on 4D-CT had higher AUC values than highly ventilated regions, suggesting that functional planning based on poorly ventilated regions may reduce the risk of lung toxicity in radiation therapy.

## Introduction

Lung cancer is the leading cause of cancer and has the highest mortality rate in Japan [1]. Functional image-based treatment planning has been investigated by many researchers for its potential to reduce lung toxicity after radiation therapy [2–5]. Single photo emission computed tomography (SPECT), positron emission computed tomography (PET), and magnetic resonance (MR) imaging have been used as functional imaging techniques for radiation therapy treatment planning; these techniques can potentially provide information on perfusion in addition to ventilation, and this may be more important than ventilation alone [2–6]. However, these functional imaging techniques require additional cost and time. As a result, functional image-based treatment planning has not become widespread in the radiation therapy community.

To address this issue, a new technique for ventilation imaging using four-dimensional computed tomography (4D-CT) with deformable image registration (DIR) has been reported [6–11]. In clinical practice, 4D-CT images are routinely acquired from thoracic cancer patients, to permit accurate contouring of gross tumor volume (GTV) with respiratory motion. 4D-CT ventilation imaging technique is referred to as a surrogate measure of ventilation. 4D-CT ventilation imaging technique can be used for treatment planning [6–9], allowing ventilation imaging to be obtained without the additional cost and time of other functional imaging modalities. The 4D-CT ventilation information can be calculated using DIR performed from the peak-exhale and peak-inhale images. In addition, quantitative analysis techniques are required to calculate the ventilation values. Currently, two calculation algorithms have been proposed: the Jacobian algorithm and the Hounsfield Unit (HU) change-based algorithm [12–13]. Differences between the ventilation images generated by from the Jacobian and HU (or density) change-based algorithms have been discussed in previous papers [12–13].

Castillo et al. reported that both Jacobian- and density-change-based methods correlate well with global measurements of resting tidal volume [12]. In addition, the correlation with clinical SPECT was evaluated using the Dice similarity coefficient, with the result that the density-change-based specific ventilation showed a statistically higher correlation (*p <*10^−4^) with the clinical reference than did the Jacobian-based implementation [13].

To compare the Xe-CT ventilation measurements to the Jacobian from the image registration transformation, for each animal we manually registered the Xe-CT slices to a 3D rectangular region in the 0 cm H2 O airway pressures image by using a rigid transformation to match major anatomic landmarks [14]. There are low correction with comparing CT ventilation and Xe-CT from sheep [15].

They concluded that using 3D image registration to match images acquired at 10-cm H_2_O and 15-cm H_2_O airway pressures gave the best match between the average Jacobian and the xenon CT-specific ventilation (linear regression, average r^2^ = 0.73) [14]. Yamamoto et al. reported on a protocol developed for a prospective clinical trial to investigate the safety and feasibility of CT ventilation functional image−guided radiation therapy [16]. Moreover, CT ventilation functional image-guided radiation therapy plans were designed to minimize the specific lung dose–function metrics, including functional V20 (fV20), while maintaining target coverage and meeting standard constraints for other critical organs [17]. Ireland et al. reported on the application of MR, SPECT, PET and CT-based measures of lung biomechanics, reviewing the practical issues involved with implementation of lung avoidance, including image registration and the role of both ventilation and perfusion imaging [6]. In these studies highly ventilated regions were avoided according to the CT ventilation functional image-guided radiation therapy plans. The use of 4D-ventilation to compare poorly and highly ventilated regions may help to further minimize pulmonary toxicity.

In this study, 4D-ventilation imaging was used to separate poorly and highly ventilated regions. Moreover, we evaluated correlations between 4D-CT ventilation-based dosimetric parameters and the clinical outcomes of thoracic cancer patients treated with radiation therapy.

## Materials and methods

All patients provide informed consent to participate in this study. The institutional Research Ethics Board at Kindai University Hospital (Osaka, Japan) Gave approval for their participation in this study (No. 28–113).

### Patient characteristics

This prospective study was approved institutional review board. Forty thoracic cancer patients who received conventionally-fractionated or hypo-fractionated radiation therapy were included. Fifteen patients were treated with conventional radiation therapy, six were treated with hyperfractionated radiation therapy and 19 patients were treated with stereotactic body radiation therapy (SBRT). Risk factors for lung disease, pretherapeutic lung disease, infectious lung disease during or after therapy and systemic therapy during and after radiotherapy were analyzed. In this study, infectious lung disease during therapy was not founded. In this study, risk factors for lung disease, pretherapeutic lung disease were defined chronic obstructive pulmonary disease, interstitial pneumonia, emphysema and lung resection. All patients were treated in our hospital from October 2011 to March 2015. The patients’ characteristics are given in Table 1.

**Table 1:** Patients’ background.

Characteristics			Value or number	
Gender				
No. of males			30	
No. of females			10	
Median age (range)			66 (57–87)	
Patients, treatment characteristics + Irradiation technique				
Conventional RT			15	
Hyper fractionated RT			6	
Stereotactic body RT			19	
Dose prescription				
Conventional RT			60–66 Gy/30-33 fr	
Hyperfractionated RT			45–54 Gy/30-36 fr	
Stereotactic body RT			52 Gy/4 fr	
Median follow up period (range)			18 months (6–48 months)	
Radiation pneumonitis				
Grade ≥2			10 (Conventional RT; 5, Hyperfractionated RT; 2, SBRT; 3)	
Grade 1			30 (Conventional RT; 10, Hyperfractionated RT; 4, SBRT; 16)	
Systemic therapy			18 (Grade ≥2; 5, Grade1; 13)	
Risk factor for pretherapeutic lung disease			20 (Grade ≥2; 5, Grade 1; 15)	
Infectious lung disease after therapy			3 (Grade 1)	

Radiation pneumonitis was graded according to the Common Terminology Criteria for Adverse Events (CTCAE) version 4.0. Patients with written informed consent were followed up at 1 and 3 months after treatment completion, and then every 3 months for 5 years according to the Kindai protocol.

### 4D-CT ventilation

The 4D-CT scans were performed using an Optima CT (GE Medical Systems, Waukesha, WI) in cine mode with the Varian Real-time Position Management (RPM) system (Varian Medical Systems, Palo Alto, CA). The scans were acquired with a 2.5 mm slice thickness. A phase-based sorting pattern was used for the 4D-CT images was used. In this phase-based sorting, the GE Advantage 4D software (GE Medical Systems) was used to create a 4D-CT image set by sorting CT slices into 10 respiratory bins, according to the RPM phase information. The next step was to use DIR for spatial mapping of the peak-exhale 4D-CT images to the peak-inhale images to derive a displacement vector field. The B-spline-based DIR algorithm implemented in iVAS (ITEM Corporation, Japan) was used for this mapping [18], with the geometrical accuracy of this algorithm having been previously validated [18–19]. Participants calculate deformation fields and submit them to the EMPIRE10 organizational team for independent evaluation. The deformation fields are evaluated over four individual categories: lung boundary alignment, fissure alignment, correspondence of manually annotated point pairs, and the presence of singularities in the deformation field [19].

We didn’t used ventilation weighted measure. We chose this DIR algorithm because previous studies had shown it to have the most accurate results for thoracic imaging [20–21]. The 4D-CT based ventilation images were then created using the Jacobian metric [13,22]. In addition, we normalized each ventilation map by converting it to percentile images.

### Ventilation-based dosimetric parameters

The ventilation-based dosimetric parameters of Mean Dose, V5, and V20 were calculated in highly and poorly ventilated regions. Previous researchers have reported a correction with between radiation pneumonitis and the ventilation-based dosimetric parameters of Mean Dose, V5, and V20 [23–24]. The conventional RT, hyperfractionated RT, and SBRT were reanalyzed to estimate the normalized total dose (NTD) according to the following equation [25–27],

NTD=D(1+dαβ1+2αβ)(Gy)Disthetotaldoseanddisthefractiondose,αβis3. (1)

The venous oxygen saturation of all patients was also investigated, both before and after radiation therapy.

To calculate the ventilation-based dosimetric parameters for the highly ventilated regions, the lung regions were evaluated according to the following percentile ventilation ranges; 90%–100%, 80%–100%, 70%–100%, 60%–100%, 50%–100%, 40%–100%, 30%–100%, 20%–100%, 10%–100%, and 0–100% where 0–100% means the total lung region. Similarly, to calculate the ventilation-based dosimetric parameters for the poorly ventilated regions, lung regions were evaluated according to the following percentile ventilation ranges; 0–10%, 0–20%, 0–30%, 0–40%, 0–50%, 0–60%, 0–70%, 0–80%, 0–90%, and 0–100%.

### Analysis

To test whether the ventilation-based dosimetric parameters could be used to predict radiation pneumonitis of ≥Grade 2 or higher, the area under the curve (AUC) was determined from receiver operating characteristic (ROC) analysis [4, 28–29]. Using the AUC value with the ROC and logistic regression analysis, radiation pneumonitis of ≥Grade 2 or higher was represented by positive values, while Grade 1 radiation pneumonitis was represented by negative values. This analysis was performed using XLSTAT-software (Addinsoft, Paris, France) [30]. Student’s *t*-test and logistic regression were used to compare the dosimetric parameters from highly ventilated regions with those from poorly ventilated regions over various percentile ventilation ranges (e.g., 0–10% for poorly ventilated regions vs. 90%–100% for highly ventilated regions). Statistical significance was defined as *p* < 0.05.

## Results

With a median follow-up duration of 18 months (range, 6–48 months), radiation pneumonitis of Grade 2 or above was observed in 10 patients: 7 patients with Grade 2 pneumonitis, two patients with Grade 3, and one patient with Grade 5. The remaining patients had Grade 1 radiation pneumonitis. In this study, there were no significant correlation between radiation pneumonitis (Grade ≥2) and pretherapeutic lung disease, infectious lung disease after therapy or systemic therapy during and after radiation therapy.

**Fig 1:**
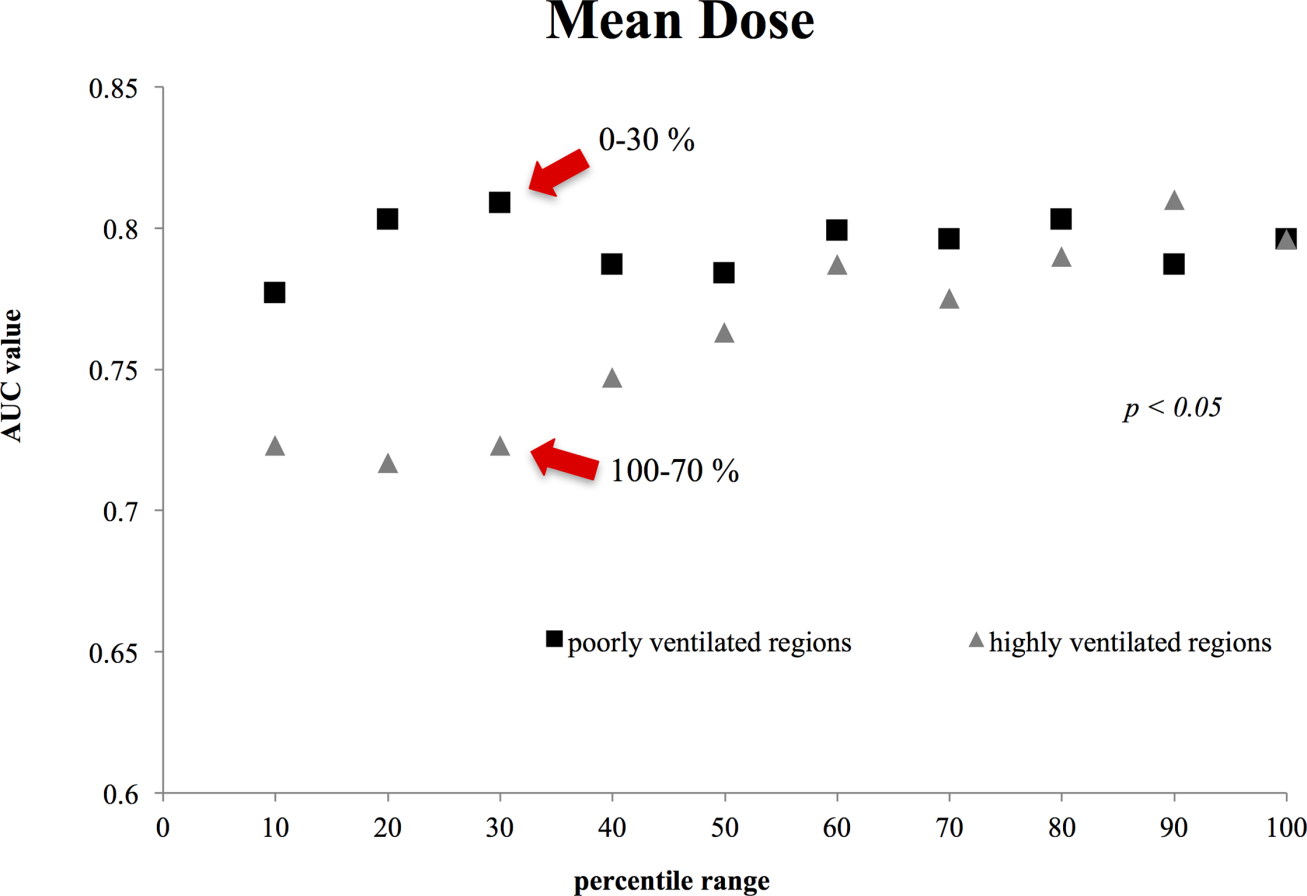
Comparison between the Mean Dose AUC values for highly and poorly ventilated regions. The difference in mean dose between poorly and highly ventilated regions was statically significant for each percentile range (*p* = 0.0093; Student’s *t*-test).

**Fig 2:**
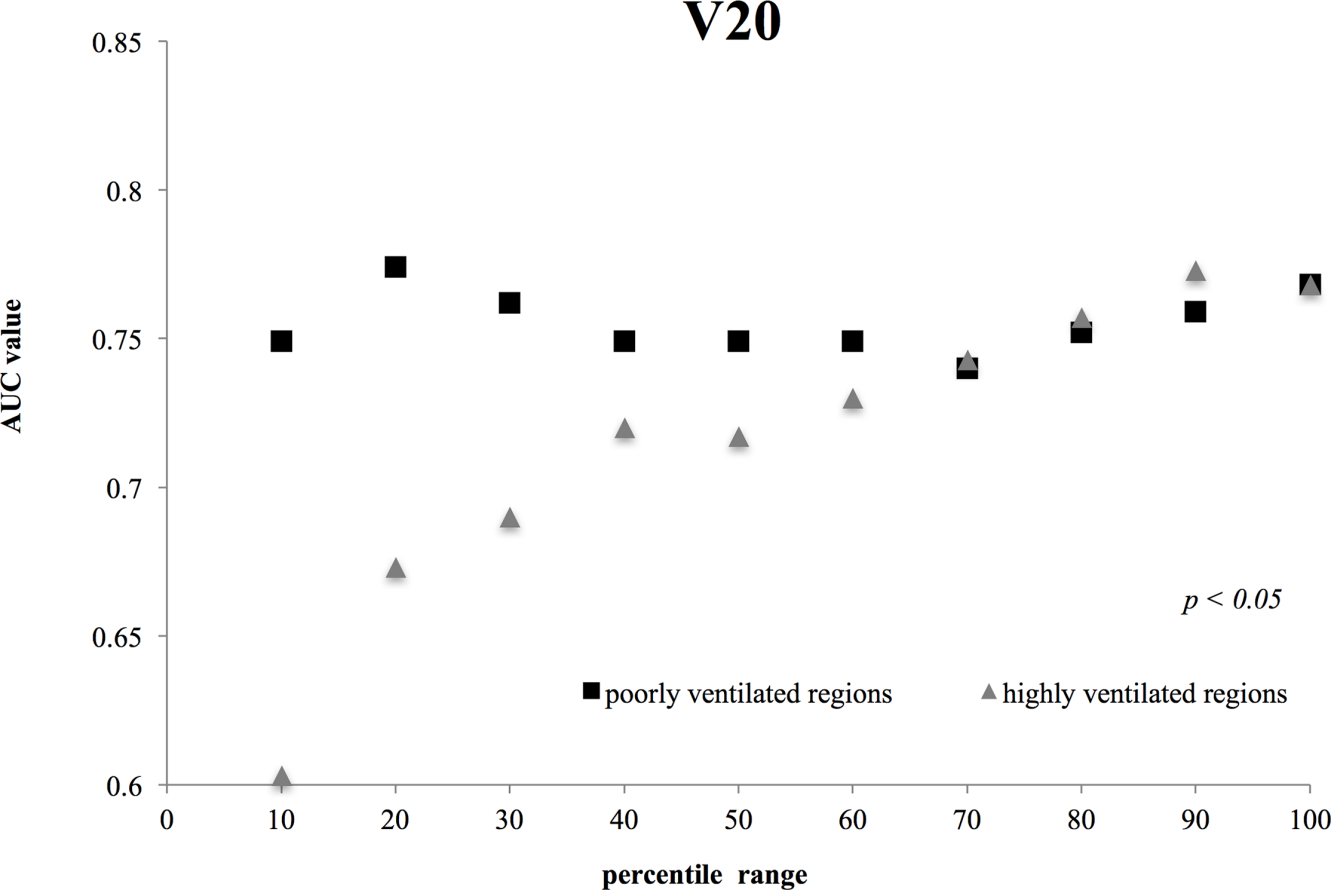
Comparisons between the V20 AUC values for highly and poorly ventilated regions. The difference in V 20 between poorly and highly ventilated regions was statically significant for each percentile range (*p* = 0.0138; Student’s *t*-test).

**Fig 3:**
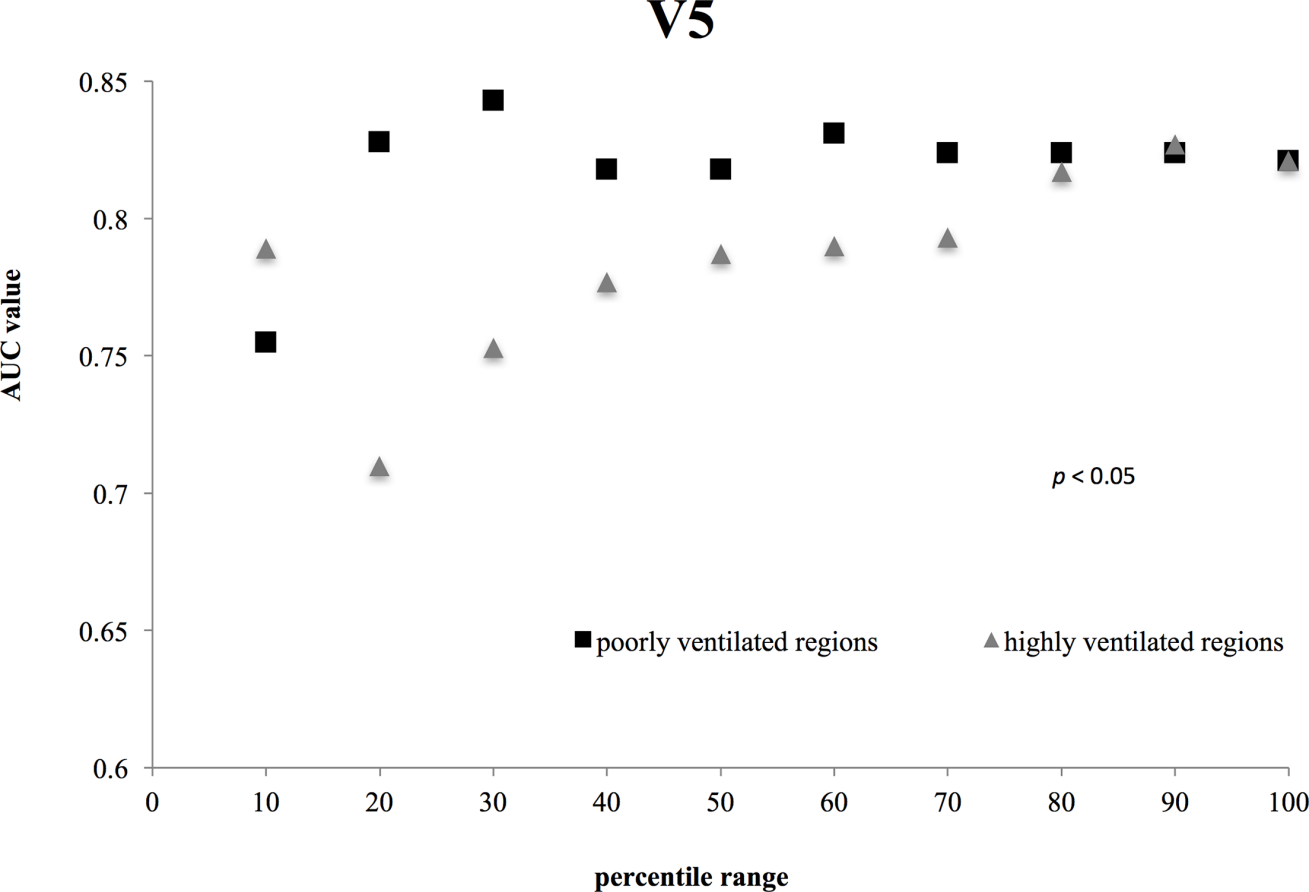
Comparisons between the V5 AUC values for highly and poorly ventilated. The difference in V 5 between poorly and highly ventilated regions was statically significant for each percentile range (*p* = 0.0236; Student’s *t*-test).

Figs 1–3 show the AUC values for Mean Dose (Fig 1), V20 (Fig 2), and V5 (Fig 3) for each ventilated lung region. In these figures, it should be noted that the x-axis represents different ranges for highly and poorly ventilated regions. For example, when the x value is 30, the plot for the highly ventilated region means that the AUC value is for the lung region with a 70–100 percentile ventilation range. However, the plot for the poorly ventilated region means that the AUC value is for the lung region with 0–30 percentile ventilation range. For all of the dosimetric parameters, the highest AUC values were observed for poorly ventilated regions (e.g., the 30% range for Mean Dose with the value of 0.809 (95% CI, 0.663 to 0.955), the 20% range for V20 with the value of 0.774 (95% CI, 0.598 to 0.915), and the 30% range for V5 with the value of 0.843 (95% CI, 0.732 to 0.954)). In addition, for highly ventilated regions, the AUC value increased with increasing percentile range.

Figs 1–3 demonstrate significant differences in all dosimetric parameters between poorly and highly ventilated regions for each percentile range (*p* < 0.05; Student’s t test).

Table 2 summarizes of the dosimetric parameters for highly ventilated regions, poorly ventilated regions, and the total lung for two patient sub-groups: one group with radiation pneumonitis of ≥Grade 2, and the other group with radiation pneumonitis of Grade 1. In this analysis, we defined the highly ventilated region as the 70%–100% ventilation value and the poorly ventilated region as the 0–30% value. For all of the dosimetric parameters, the group with radiation pneumonitis of ≥Grade 2 had higher values than the group with radiation pneumonitis of Grade 1 (e.g., V20 in highly ventilated region, 20.9 ±17.5% vs. 10.4 ±12.2%). In the group with radiation pneumonitis of ≥Grade 2, all dosimetric parameters in the poorly ventilated regions had higher values that those in the highly ventilated regions.

**Table 2:** Summary of the dosimetric parameters calculated for highly ventilated regions, poorly ventilated regions, and total lung for two patient sub-groups: One with radiation pneumonitis of ≥Grade 2 and the other with radiation pneumonitis of Grade 1.

Radiation Pneumonitis	Dosimetric	Poorly ventilated regions	Highly ventilated regions	Total lung
	Parameter	(0–30%)Average, SD	(70–100%) Average, SD	(0–100%) Average, SD
≥ Grade 2 (n = 10)	Mean Dose (Gy)	19.1±16.3	15.6±7.5	14.4±6.4
	V 20 (%)	29.6±15.8	20.9±17.5	20.0±12.0
	V 5 (%)	44.8±19.3	37.5±16.3	38.9±8.9
< Grade 2 (n = 30)	Mean Dose (Gy)	9.8±6.7	12.3±7.3	11.7±11.2
	V 20 (%)	6.6±6.6	10.4±12.2	9.4±10.0
	V 5 (%)	24.4±16.8	26.4±13.0	25.6±11.8

Table 3 summarizes of the dosimetric parameters for highly ventilated regions and poorly ventilated regions for the two patient sub-groups. There were significant differences in all dosimetric parameters between Grade 1 and Grade >2, in both for comparison of poorly and highly ventilated regions.

**Table 3:** *P*-values for *t*-tests and logistic regression comparing Grade 1 and ≥ Grade 2 radiation pneumonitis in both poorly and highly ventilated regions.

Variation	*p*-Value	*p*-Value
	(t-test)	(logistic regression)
*Grade 1 vs*. *≧ Grade 2*		
Poorly ventilation (30th percentile range)		
Mean Dose	0.0487	0.0462
V 5	0.0006	0.2669
V 20	0.0070	0.0150
Highly ventilation (70 th percentile range)		
Mean Dose	0.1118	0.6458
V 5	0.0438	0.7195
V 20	0.0486	0.9839
total lung		
Mean Dose	0.1752	0.5724
V 5	0.0006	0.2003
V 20	0.0086	0.5286

**Fig 4:**
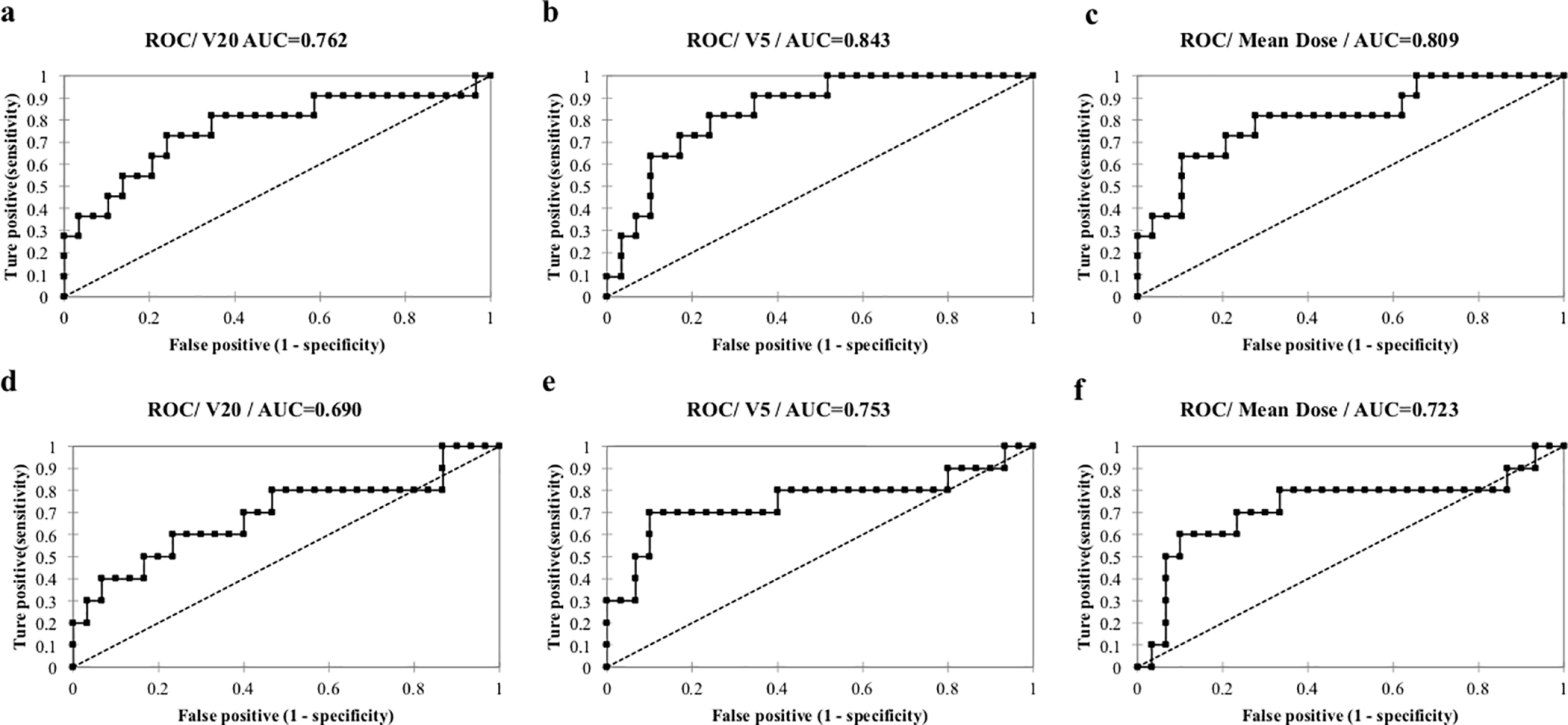
**Representative ROCs from 6 cases; for poorly ventilated regions:** (a) Mean Dose (b) V5, (c) V20, and for highly ventilated regions: (d) Mean Dose (e) V5, (f) V20.

Fig 4 shows representative ROC curve results (6 cases; Mean Dose, V5, V20, with poorly and highly ventilated regions shown for each parameter).

**Fig 5:**
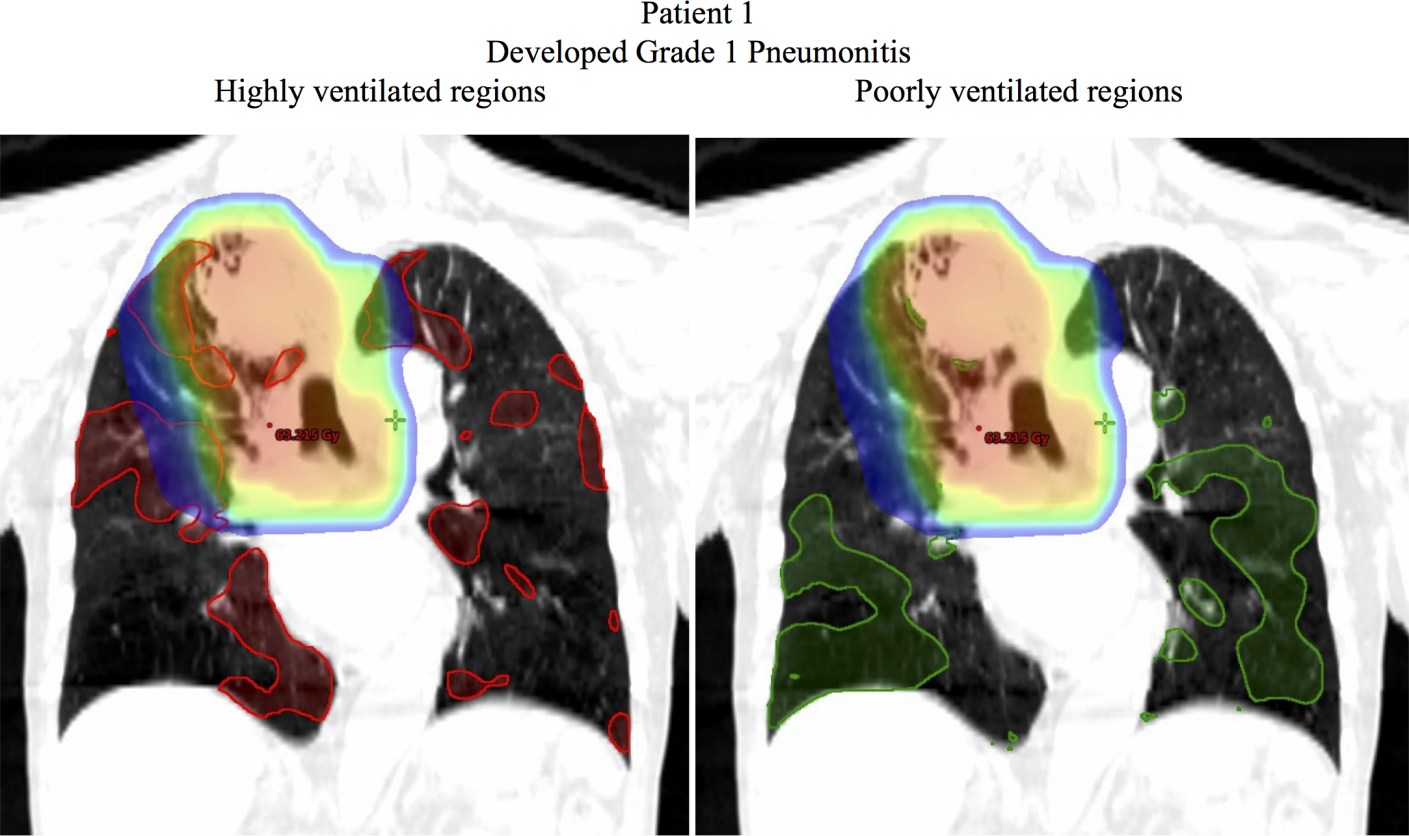
Example case of a patient without severe radiation pneumonitis (Grade 1). The red contour indicates the highly ventilated region (70th–100th percentiles) and the green contour indicates the poorly ventilated region (0–30th percentiles). The dose >5 Gy is shown.

**Fig 6:**
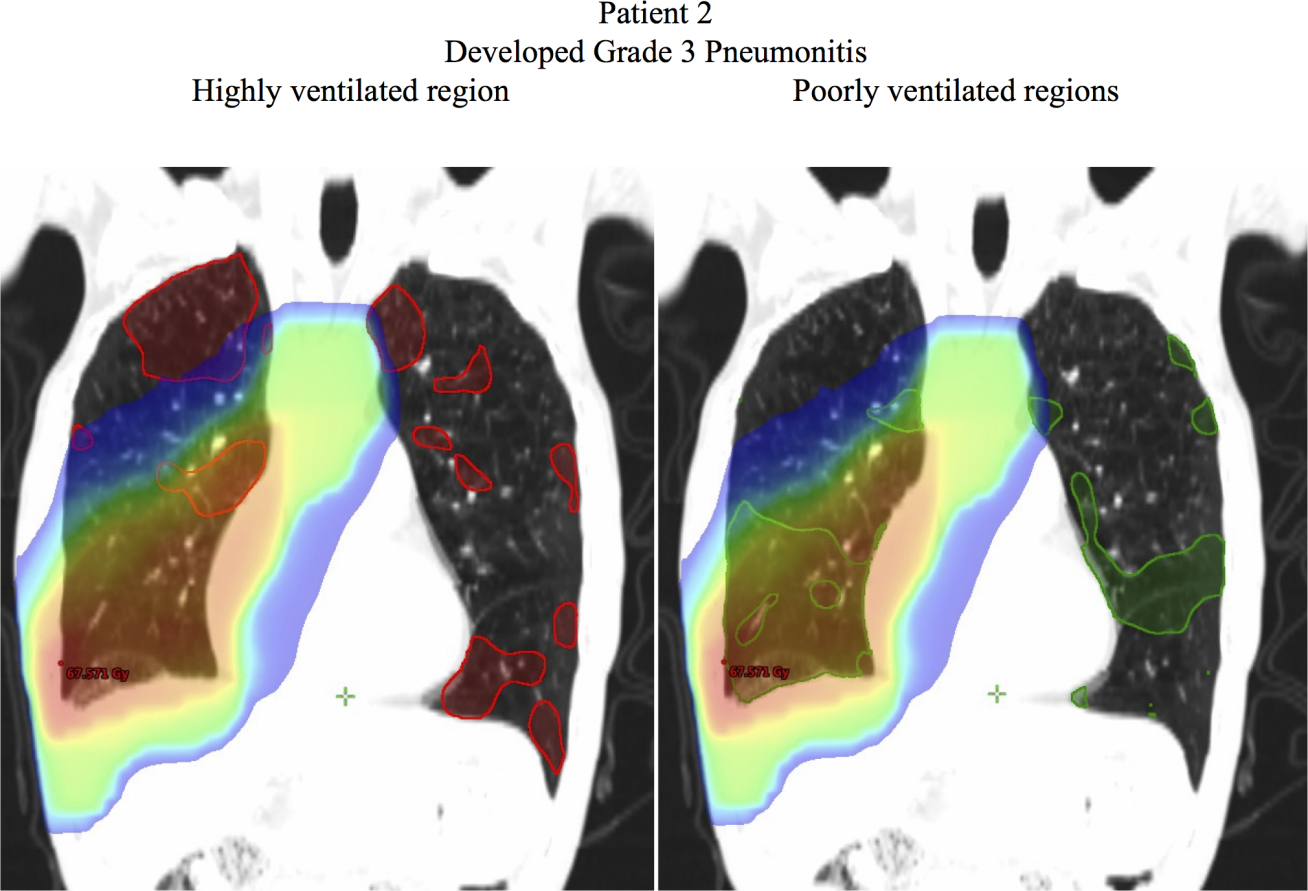
Example case of a patient with severe radiation pneumonitis (Grade 3). The red contour indicates the highly ventilated region (70th–100th percentiles) and the green contour indicates the poorly ventilated region (0–30th percentiles). The dose >5 Gy is shown.

**Fig 7:**
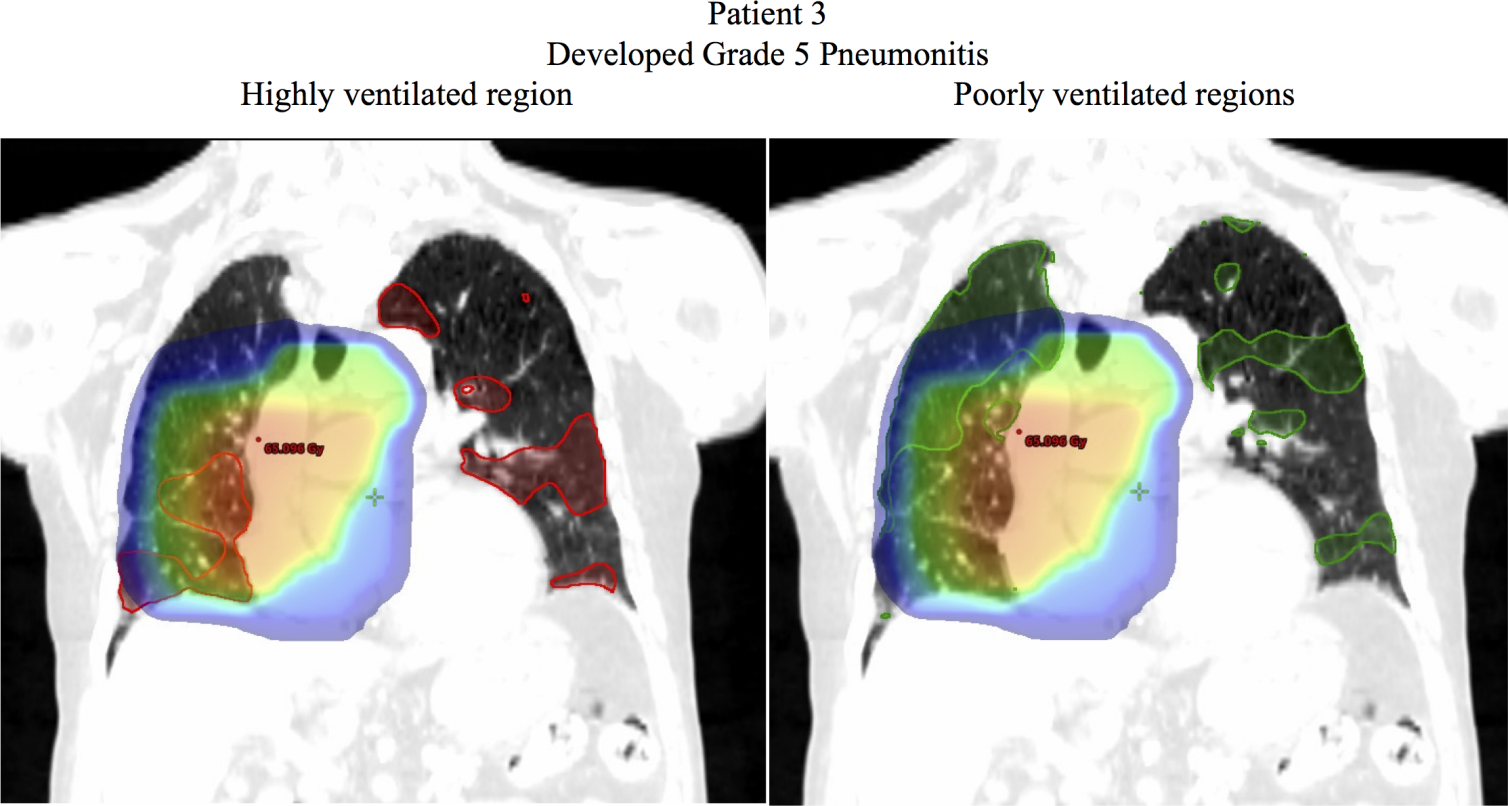
Example case of a patient with severe radiation pneumonitis (Grade 5). The red contour indicates the highly ventilated region (70th–100th percentiles) and the green contour indicates the poorly ventilated region (0–30th percentiles). The dose >5 Gy is shown. In this patient, there was no clear association between the ventilation-based dosimetric parameters and the ventilation state of the region.

Figs 5–7 show examples of dose distributions overlaid with the highly and poorly ventilated regions. Fig 5 shows an example case of a patient without severe radiation pneumonitis (Grade 1). The Mean Dose, V20, and V5 for the total lung were 10.7 Gy, 17.4%, and 30.1% respectively. For the highly ventilated region, these parameters in the highly ventilated region were 15.1 Gy, 25.5%, and 42.8% respectively, whereas for the poorly ventilated region, they were 5.4 Gy, 6.0%, and 12.6%. This clearly indicated that all the dosimetric parameters had lower values in the poorly ventilated region than in the highly ventilated region, and that according to visual inspection, this patient received a higher dose to the highly ventilated region. Conversely, Fig 6 shows an example case of a patient with severe radiation pneumonitis (Grade 3). Mean Dose, V20, and V5 for the total lung were 17.8 Gy, 29.2%, and 43.6%, respectively. For the highly ventilated region these parameters were 15.1 Gy, 25.5%, and 42.8% respectively, whereas for the poorly ventilated region they were 28.3 Gy, 47.5%, and 59.2%. This showed that the poorly ventilated region had a higher value in all the dosimetric parameters than in the highly ventilated region, and that according to visual inspection, this patient received a higher dose to the poorly ventilated region. Fig 7 shows an example case of a patient with severe radiation pneumonitis (Grade 5). The Mean Dose, V20, and V5 for the total lung were 19.5 Gy, 34.1%, and 47.2% respectively. In the highly ventilated region these parameters were 17.7 Gy, 30.9%, and 45.1% respectively, whereas in the poorly ventilated region they were 21.4 Gy, 37.1%, and 50.2%. This suggests that there was no correlation between ventilation-based dosimetric parameters and lung toxicity.

Finally, for the entire patient group, there was no significant differences in the venous oxygen saturation before and after radiation therapy.

## Discussion

In this study, we aimed to clarify the relationship between 4D-CT ventilation-based dosimetric parameters and clinical outcomes. Yamamoto et al. previously employed 4D-CT ventilation-based functional planning for lung cancer patients to reduce the dose to highly ventilated regions [15]. Furthermore, Vinogradskiy et al. demonstrated that incorporating ventilation-based functional imaging improved the prediction of radiation pneumonitis, although the results were not significant at the 0.05 level when compared with other methods not using ventilation-based functional imaging [6]. Our results were statistically significant for the AUC value of poorly ventilated regions, and suggest the potential of 4D-CT ventilation-based functional planning for a reducing the risk of lung toxicity after radiation therapy. However, our results were the opposite of previous studies. On the basis of Figs 1–3, our results suggest that dose to poorly ventilated regions (0–30%) was associated with lung toxicity risk, and the data in Table 3 suggest that the dose to poorly ventilated regions (0–30%) may reduce the lung toxicity risk. Additionally, the highest AUC values for all dosimetric parameters were observed in the poorly ventilated regions (0–30%, 0–20%). As the percentile range for highly and poorly ventilated regions increased, the AUC values approached those of the total lung. Thus, the AUC value for 10% showed fluctuation for evaluating narrow lung area.

Aibe et al. demonstrated that lung V5 may be correlated with Grade 5 radiation pneumonitis [31]. On the basis of our results, we could possibly explain this grade 5 radiation pneumonitis according to the ventilation-based dosimetric parameters. When a low dose is extended to poorly ventilated regions, Grade 5 radiation pneumonitis may occur, even though the V5 value is not large.

Several factors may influence 4D-CT ventilation imaging, including the DIR accuracy and the reproducibility of the patient’s respiratory pattern. Yamamoto et al. showed that the choice of DIR algorithm could change the results of 4D-CT ventilation imaging [32]. Although we chose a DIR algorithm giving reasonable accuracy, it is impossible to eliminate residual DIR error. With respect to the reproducibility of the patient’s respiratory pattern during 4D-CT ventilation imaging, Du et al. clearly showed that 4D-CT ventilation had good reproducibility in anesthetized, mechanically ventilated animals, but variations in respiratory effort and breathing patterns reduced reproducibility in spontaneously breathing humans [33]. Biological rationale between radiation pneumonitis (≧ Grade 2) and highly ventilated regions has not been shown. Some reports showed that pulmonary emphysema was a high risk factor [29, 34]. Poorly ventilated regions might be high radiation sensitivity as the pulmonary emphysema.

Although we made considerable effort to reduce irregular respiratory patterns during image acquisition, there were still residual artifacts in the 4D-CT data sets used in this study. These residual errors could have reduced the accuracy of our results.

### Limitations

First, only forty patients were analyzed in this study. In addition, the study group included patients treated with conventionally-fractionated radiation therapy and hypo-fractionated radiation therapy. Finally, we did not analyze additional factors such as smoking. Smoking, risk factors for lung disease, pretherapeutic lung disease, infectious lung disease during or after therapy and systemic therapy during and after radiotherapy have been shown to be associated with risk of toxicity [34–35]. In this study, there were no significant difference in X^2^ test between radiation pneumonitis (≧ Grade 2) and risk factors such as COPD, interstitial pneumonia, systemic therapy and infections.

## Conclusions

Our results showed that dose deposition cannot reduce lung toxicity, but in contrast to highly ventilated regions, dose deposition in poorly ventilated regions might be accompanied by a reduced lung toxicity risk. In the comparisons of poorly and highly ventilated regions, there were significant differences in all dosimetric parameters between patients who developed radiation pneumonitis of Grade 1 and those who developed Grade 2 or higher. In the next study, we will conduct a prospective clinical trial to investigate the safety and feasibility of reducing the dose to areas defined as being poorly ventilated on CT ventilation image-guided radiation therapy.
