# Correction: Evaluation of lung toxicity risk with computed tomography ventilation image for thoracic cancer patients

**DOI:** 10.1371/journal.pone.0225965

**Published:** 2019-11-27

**Authors:** Masakazu Otsuka, Hajime Monzen, Kenji Matsumoto, Mikoto Tamura, Masahiro Inada, Noriyuki Kadoya, Yasumasa Nishimura

In the Results subsection of the Abstract, there are errors in the first two sentences. The correct sentences are: For Mean Dose, poorly ventilated lung regions in the 0–30% range showed the highest AUC value (0.809: 95% confidence interval [CI], 0.754−0.973). For V20, poorly ventilated lung regions in the 0–20% range had the highest AUC value (0.774: 95% [CI], 0.679−0.869), and for V5, poorly ventilated lung regions in the 0–30% range had the highest AUC value (0.843:95% [CI], 0.732–0.954).

There are errors in the fifth sentence of the second paragraph of the Results section. The correct sentence is: For all of the dosimetric parameters, the highest AUC values were observed for poorly ventilated regions (e.g., the 30% range for Mean Dose with the value of 0.809:95% [CI], 0.754−0.973, the 20% range for V20 with the value of 0.774:95% [CI], 0.679−0.869, and the 30% range for V5 with the value of 0.843:95% [CI], 0.732–0.954 ).

There is an error in the first sentence of the Patient characteristics subsection of the Materials and methods. The correct sentence is: This retrospective study was approved by institutional review board.

There is an error in the first sentence of the Conclusions. The correct sentence is: Our results showed that dose disposition cannot reduce lung toxicity in highly ventilated regions in contrast, dose disposition in poorly ventilated regions might be accompanied by a reduced lung toxicity risk.

There is an error in affiliation 1 for authors Masakazu Otsuka, Hajime Monzen, Kenji Matsumoto, and Mikoto Tamura. The correct affiliation 1 is: Department of Medical Physics, Graduate School of Medical Sciences, Kindai University, Osakasayama, Japan.

There are errors in the captions for Figs 1–4. Please see the complete, correct captions for Figs 1–4 here.

**Fig 1:**
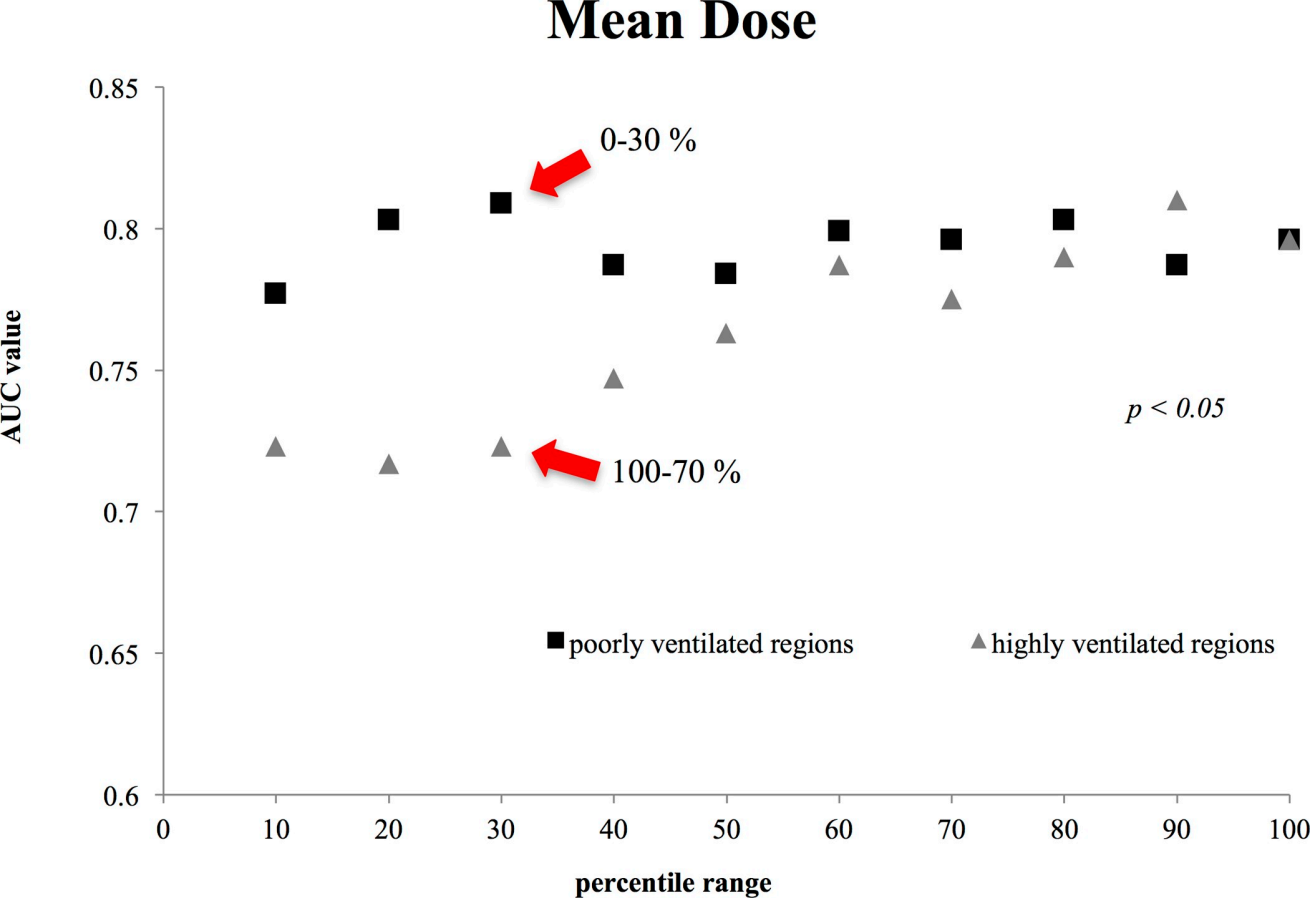
Comparison between the mean dose AUC values for highly and poorly ventilated regions. The difference in mean dose between poorly and highly ventilated regions was statically significant (*p* = 0.0093; Student’s *t*-test).

**Fig 2:**
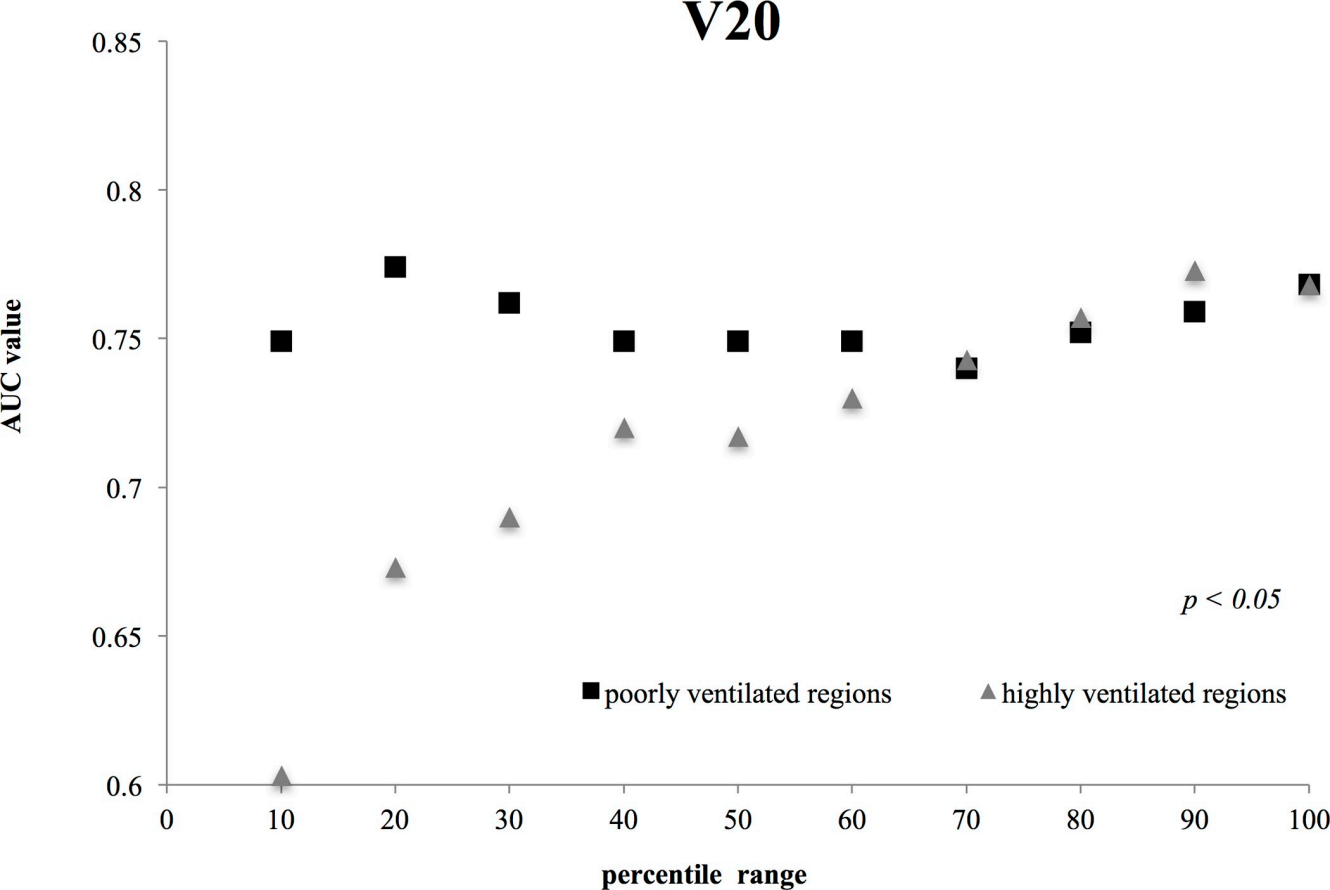
Comparisons between the V20 AUC values for highly and poorly ventilated regions. The difference in V 20 between poorly and highly ventilated regions was statically significant (*p* = 0.0138; Student’s *t*-test).

**Fig 3:**
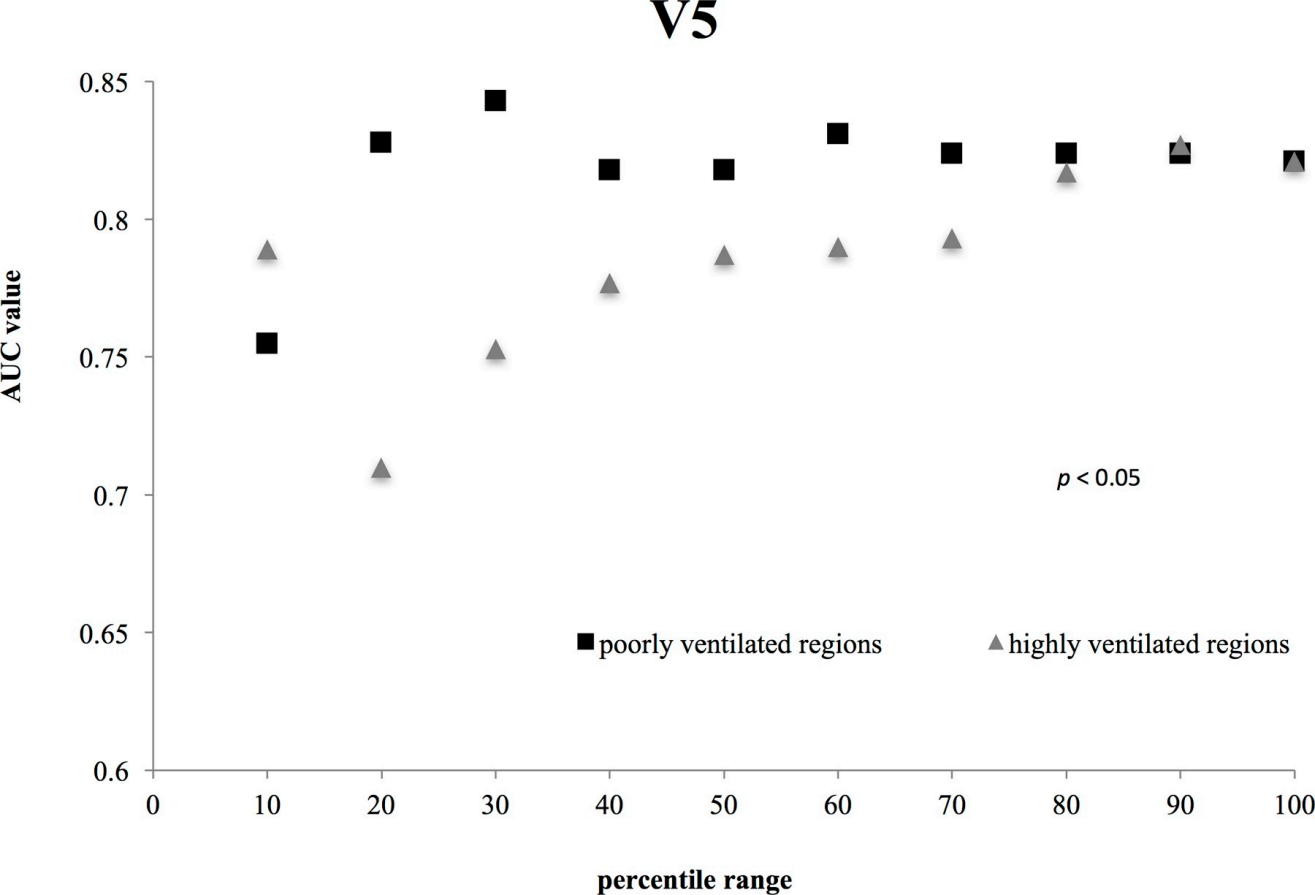
Comparisons between the V5 AUC values for highly and poorly ventilated. The difference in V 5 between poorly and highly ventilated regions was statically significant (*p* = 0.0236; Student’s *t*-test).

**Fig 4:**
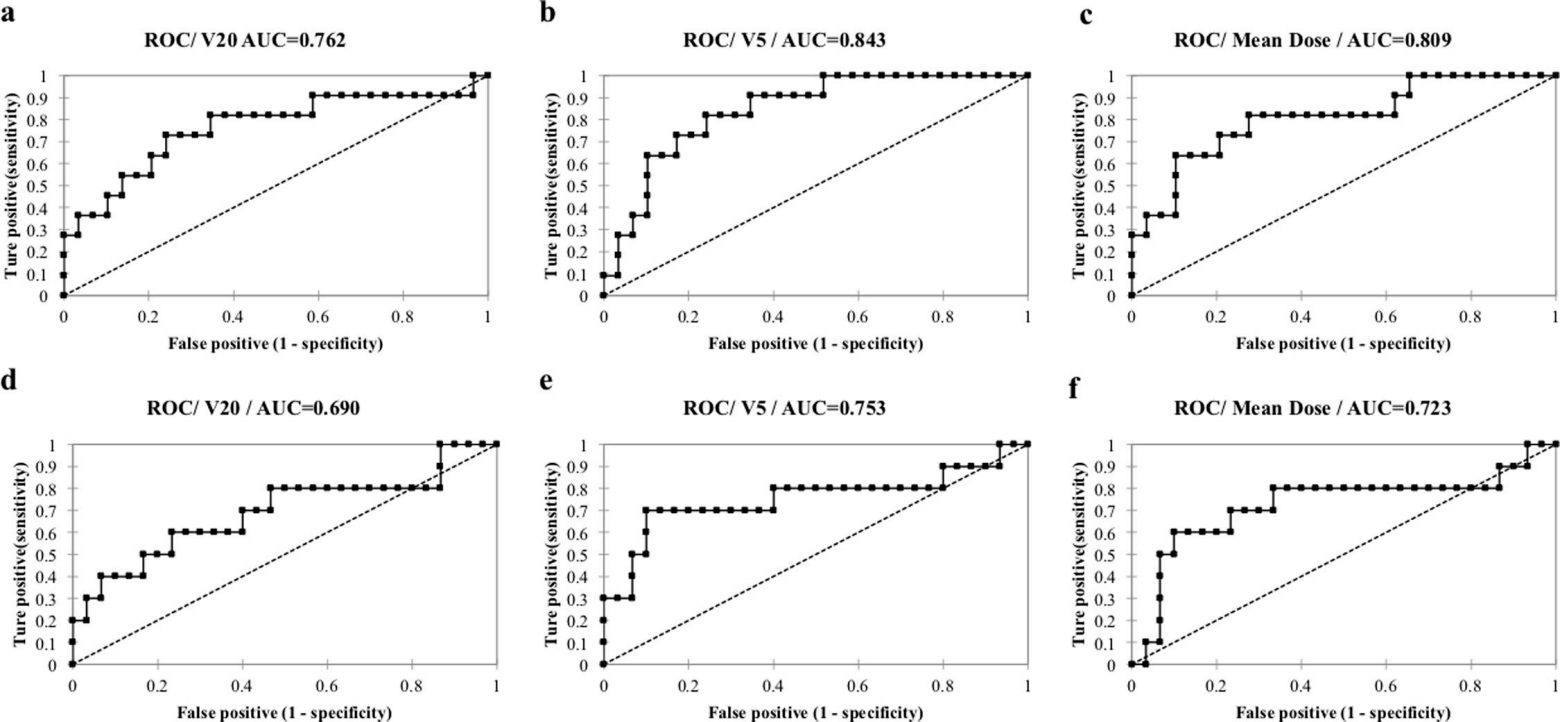
Representative ROCs from 6 cases; for poorly ventilated regions. (a) V20, (b) V5, (c) Mean Dose, and for highly ventilated regions: (d) V 20, (e) V5, (f) Mean Dose.
